# A Case Report on Bortezomib-Induced Bilateral Chalazion

**DOI:** 10.7759/cureus.10062

**Published:** 2020-08-26

**Authors:** Mahati Paravathaneni, Vihitha Thota, Sana Mulla, Rajesh Thirumaran, Yu Y Thar

**Affiliations:** 1 Internal Medicine, Mercy Catholic Medical Center, Darby, USA; 2 Hematology/Oncology, Mercy Catholic Medical Center, Darby, USA

**Keywords:** chalazion, bortezomib, multiple myeloma

## Abstract

A chalazion is a swollen bump on the eyelid that occurs when the eyelid’s oil gland clogs up. Bortezomib is an FDA-approved novel agent for the treatment of patients with multiple myeloma (MM). Recently, there have been a few case reports on the possible association between eyelid chalazion and bortezomib therapy. We describe the case of an 87-year-old female diagnosed with MM who developed bilateral eye chalazion after being initiated on bortezomib. She was evaluated by an ophthalmologist, started on eye lubricants and antibiotic eye drops, and the bortezomib was discontinued until the resolution of the lesions was achieved. Physicians should be aware of these ocular complications of bortezomib, as early intervention can prevent worsening eyelid complications.

## Introduction

Chalazion, also known as a meibomian cyst, is a type of painless, localized fluid-filled bump on the upper or lower eyelid. It is usually a benign self-limiting reaction caused by the inflammation of the blocked meibomian gland with retained secretions [1]. Patients usually present with symptoms of eyelid swelling, pain, and localized irritation. Any condition that causes meibomian gland secretions to become unusually thick and unable to exit the gland can lead to the development of a chalazion. Often, chalazion resolves without intervention within a few days to a week; however, more extensive lesions may lead to complications, such as corneal astigmatism, mechanical ptosis, and secondary infections, including cellulitis.

In recent years, agents like bortezomib have been shown to impact survival in multiple myeloma (MM) significantly [2]. Bortezomib belongs to a class of medications known as proteasome inhibitors, and it has been FDA-approved for use in both MM and mantle cell lymphoma. The extensive usage of bortezomib in MM has helped in understanding its side-effect profile better. The most common adverse effects of the medication are gastrointestinal upset, fatigue, peripheral neuropathy, thrombocytopenia, and rashes. In this report, we present a case of a bortezomib-induced chalazion in a patient with MM.

## Case presentation

Our patient was an 87-year-old female with a past medical history significant for hypertension, hyperthyroidism, and chronic kidney disease stage III, who was admitted to the hospital for evaluation of worsening fatigue and dyspnea on exertion with a loss of appetite and weight loss of 20 lbs in six months. Her physical exam was notable for conjunctival and mucosal pallor. She was found to have normocytic, hypochromic anemia with a hemoglobin of 5.8 g/dL (normal range: 12-15 g/dL) and a mean corpuscular volume (MCV) of 80.4 fL (normal range: 80-100 fL), along with a vitamin B12 level of 208 pg/mL (normal range: 190-950 pg/mL). The patient denied any signs of bleeding, and she was not on anticoagulation. She received packed red blood cell transfusions with improvement in her hemoglobin and was started on B12 supplementation. She eventually underwent an endoscopy and colonoscopy, both of which did not reveal a source of bleeding. She later presented to our outpatient hematology/oncology clinic for evaluation of the anemia, as the hemoglobin did not improve with B12 supplementation. Further workup revealed immunoglobulin G (IgG)-positive kappa monoclonal bands on immunofixation. The skeletal survey showed extensive lytic lesions throughout the axial and visualized appendicular skeleton. She was diagnosed with IgG Kappa MM [International Staging System (ISS) stage III and Revised International Staging System (R-ISS) stage III], which was confirmed by bone marrow aspiration and biopsy. The patient had underlying chronic kidney disease with a baseline creatinine of 1.8 mg/dL and creatinine clearance (CrCl) of ~10 mL/min. Given her kidney function, she was initially started on a two-drug anti-myeloma regimen and received weekly treatment with bortezomib (1.3 mg/m^2^ on days one, eight, and 15) and dexamethasone (40 mg on days one, eight, and 15); and the cycle was repeated every 21 days. The patient tolerated three cycles of treatment well, after which she started complaining of swelling and tearing of both of her eyes. She was seen by an ophthalmologist who attributed the symptoms to the chalazion of both eyelids. Bortezomib was held, and she was started on eye lubricants and topical antibiotic eye drops. Bortezomib was restarted after a three-week lapse, as she had resolution of the chalazia from ophthalmic treatment.

**Figure 1:**
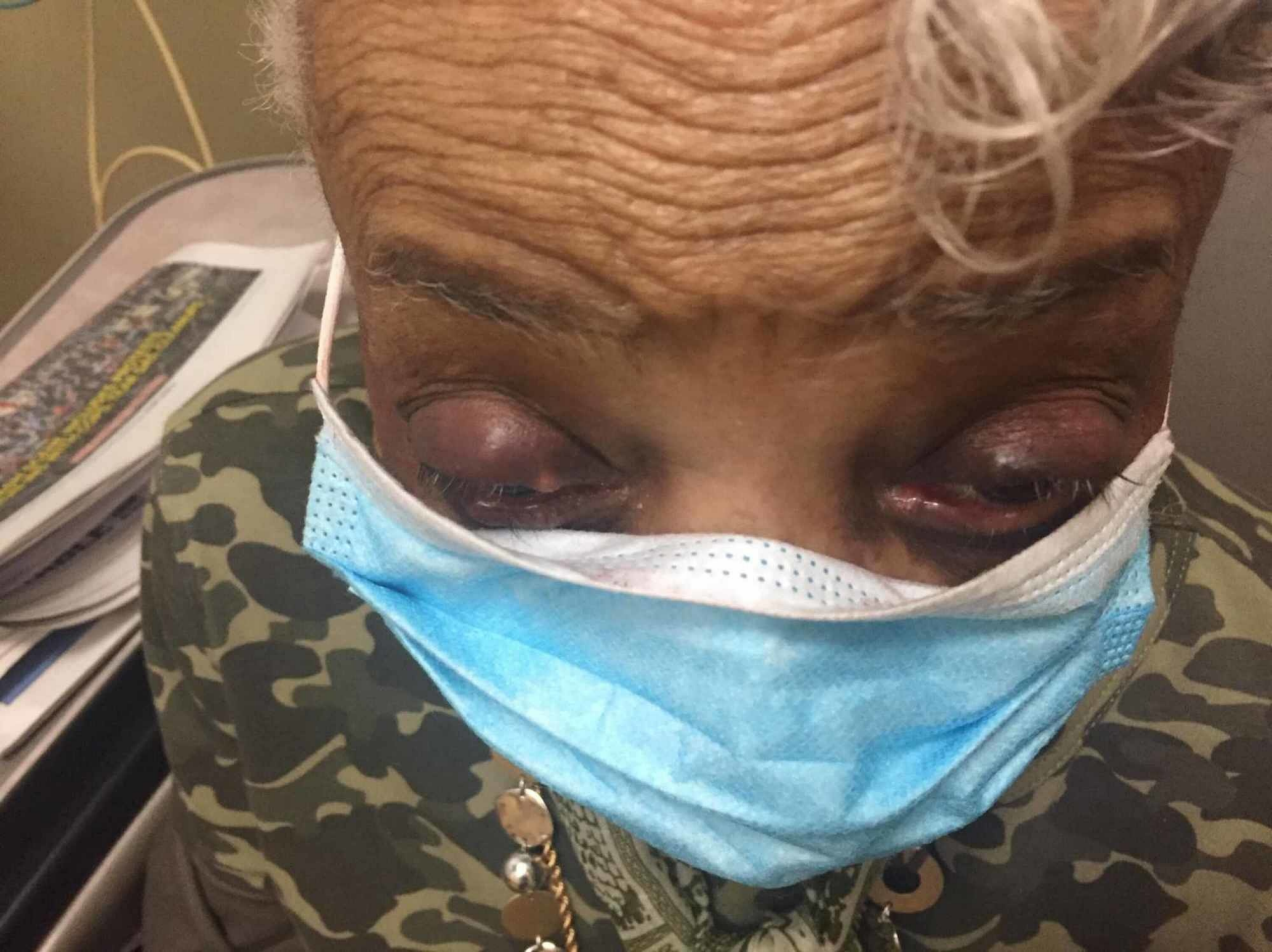
Bilateral chalazion (right more prominent than left)

**Figure 2:**
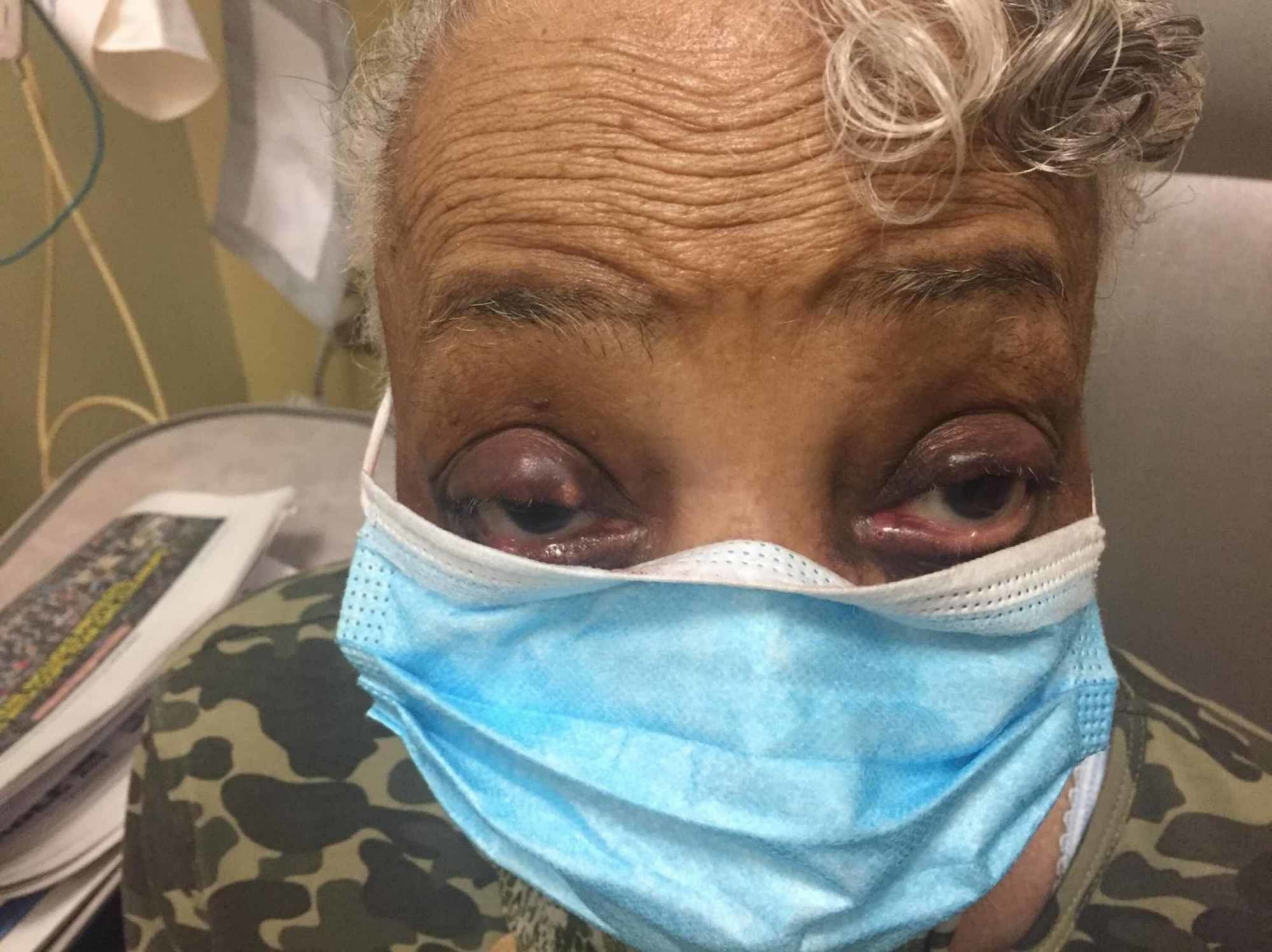
Another image showing bilateral chalazion

Figures 1, 2 show the chalazion of both the eyes; the right-eye chalazion is more prominent than the left-eye chalazion.

## Discussion

Bortezomib is a boronic acid-derived proteasome inhibitor and a first-in-class selective inhibitor of the 26S proteasome, an enzyme complex involved in protein degradation. Its anti-myeloma effect is mainly due to inhibition of endoplasmic reticulum-dependent degradation of excessively synthesized abnormal myeloma proteins [2]. It is an FDA-approved medication for primary, refractory, or relapsed cases of MM when used as a single agent or in combination with other anti-myeloma agents [3]. It is often given in combination with dexamethasone as it is postulated that this combination has a synergistic effect, with an improved response rate seen with the combination of the two versus bortezomib alone.

The adverse effects of bortezomib include gastrointestinal symptoms (nausea, vomiting, diarrhea, constipation), fatigue, peripheral neuropathy, thrombocytopenia, fever, hypotension, pain, headache, and dehydration [4]. There have also been reported cases (8-18%) of skin lesions of pruritic rash due to bortezomib. Ocular side effects of bortezomib are rare, and recent studies have shown a possible association between eyelid chalazion and bortezomib therapy [5,6]. Also, it has been noted that patients with impaired kidney function are at a higher risk of experiencing adverse effects of treatment [7]. Therefore, patients with renal impairment should be more closely monitored for possible toxicities and managed accordingly.

Most patients experience the development of chalazion around three months after the initiation of therapy [8]. The mechanism by which bortezomib causes chalazion is still unknown. It is postulated that it creates a systemic inflammatory response potentiating the release of proinflammatory cytokine interleukin 6 (IL-6), tumor necrosis factor (TNF), and C-reactive protein (CRP). A chalazion often resolves within a few months after the cessation of treatment, and there have been a few cases where the ocular symptoms have recurred with the resumption of therapy [4]. Treatment mostly includes cessation of chemotherapy until the improvement of chalazion, and conservative measures such as lid hygiene and warm compressions to facilitate drainage. Topical or intralesional steroids and oral doxycycline can be used in refractory cases; however, incision and curettage is the definitive treatment for cases not responding to conservative management.

## Conclusions

A chalazion is a rare complication of bortezomib. Clinicians must keep in mind the ocular side effects of the drug and refer the patients early on for ophthalmology evaluation due to the risk of local complications. These lesions can often be refractory to conservative management and require the discontinuation of bortezomib until symptoms improve. Early detection and intervention can prevent catastrophic eye complications.
